# Rodent Models of Depression: Neurotrophic and Neuroinflammatory Biomarkers

**DOI:** 10.1155/2014/932757

**Published:** 2014-06-05

**Authors:** Mikhail Stepanichev, Nikolay N. Dygalo, Grigory Grigoryan, Galina T. Shishkina, Natalia Gulyaeva

**Affiliations:** ^1^Laboratory of Functional Biochemistry of Nervous System and Laboratory of Conditioned Reflex and Emotion, Institute of Higher Nervous Activity and Neurophysiology, RAS, 5a Butlerov Street, Moscow 117485, Russia; ^2^Laboratory of Functional Neurogenomics, Institute of Cytology and Genetics, Russian Academy of Sciences, 10 Academician Lavrentyev Avenue, Novosibirsk 630090, Russia; ^3^Department of Physiology, Novosibirsk State University, 2 Pirogov Street, Novosibirsk 630090, Russia

## Abstract

Rodent models are an indispensable tool for studying etiology and progress of depression. Since interrelated systems of neurotrophic factors and cytokines comprise major regulatory mechanisms controlling normal brain plasticity, impairments of these systems form the basis for development of cerebral pathologies, including mental diseases. The present review focuses on the numerous experimental rodent models of depression induced by different stress factors (exteroceptive and interoceptive) during early life (including prenatal period) or adulthood, giving emphasis to the data on the changes of neurotrophic factors and neuroinflammatory indices in the brain. These parameters are closely related to behavioral depression-like symptoms and impairments of neuronal plasticity and are both gender- and genotype-dependent. Stress-related changes in expression of neurotrophins and cytokines in rodent brain are region-specific. Some contradictory data reported by different groups may be a consequence of differences of stress paradigms or their realization in different laboratories. Like all experimental models, stress-induced depression-like conditions are experimental simplification of clinical depression states; however, they are suitable for understanding the involvement of neurotrophic factors and cytokines in the pathogenesis of the disease—a goal unachievable in the clinical reality. These major regulatory systems may be important targets for therapeutic measures as well as for development of drugs for treatment of depression states.

## 1. Introduction

Depression, one of the most prevalent and life-threatening forms of mental illness affecting about 21% of the world's population, is believed to be related to individual alterations of a complex signaling network including the hypothalamic-pituitary-adrenal axis; the production of neurotrophins and proinflammatory cytokines and these alterations may be intimately involved in major mood changes. Indeed, during the progress of depression, multiple molecular, cellular, structural, and functional changes occur in the brain. Neurons and glial cells respond to these changes adaptively by employing various mechanisms in order to maintain the integrity of the brain. Preclinical and clinical studies on depression highlighted an increased production of proinflammatory markers. Though stress-induced cytokine production is adaptive at the first stage, later it becomes an important link to pathology development. According to the cytokine hypothesis, depression is caused by a stress-related increased production of proinflammatory cytokines that induce oxidative and nitrosative brain damage, impairing serotonin (5-HT) system and contributing to the glucocorticoid resistance [1]. All these factors affect neurogenesis in brain regions involved in depression and are functionally interconnected so that initial alteration in one of them results in abnormalities in the others [2].

Some authors regard depression as a disease of abnormal trophic support [3]. Neurotrophic factors (NTFs), along with cytokines, play an important role in supporting brain equilibrium in stressogenic situations and are central to many aspects of the nervous system function. Indeed, according to a modern classification, important cytokines involved in neuroinflammatory processes (IL-6, TNF-*α*) are members of neurokine superfamily belonging to NTFs [3]. These systems regulate the development, maintenance, and survival as well as the demise of neurons and glial cells. A vast amount of evidence indicates that alterations in levels of NTFs and cytokines, as well as of their receptors, can modify normal neuronal function and even lead to neuronal death.

Mounting evidence indicates that inflammatory cytokines and other intertwined pathways including neurotrophins contribute to the development of depression in both medically ill and medically healthy individuals [4, 5]. Since a number of studies have shown links between inflammatory cytokines and mood disorders, many authors regard depression as an inflammatory condition, while activated glial cells, both as source and as target of inflammatory molecules, are regarded as a potential pathophysiological target for treatment of depression [6]. However, in spite of depression prevalence in the general population and the widespread acceptance of its biological basis, progress in providing disease biomarkers or approved diagnostic tests is amazingly slow. Studies using strategies like genome-wide association and candidate gene analyses have identified a number of possible biomarkers of depression, including serum levels of neurotrophic factors, inflammatory cytokines, and HPA axis hormones, but so far none have proven sufficiently powerful for clinical use [7, 8].

Animal models are widely used as an important tool for understanding of a complex network formed by interrelationships between NTFs and cytokines in pathogenesis of depression. What should animal, specifically rodent, models of depression model? Responding to this knotty question, Frazer and Morilak [9] stressed that rather than trying to recreate or mimic the entire spectrum of symptoms comprising the syndrome of depression, it may be more informative to develop animal models for specific behavioral dimensions. This gives a hope to understand not only the neurobiological changes underlying respective symptoms but also the molecular and cellular regulatory mechanisms by which antidepressants can alleviate those symptoms. Asking which molecular mechanisms we should be attempting to study first in animal models of depression, we have in mind pathogenetically valid targets. Most of them are quite specific and include specific brain regions (e.g., HPA-related brain structures) and specific molecules (e.g., monoamine transporters, monoamine oxidase). However, there are major regulatory mechanisms controlling normal brain plasticity and, to a significant extent, forming a determinative background for specific pathological events accompanying mental diseases. These mechanisms include, in particular, the complex system of NTFs and their receptors as well as multifaceted neuroinflammatory processes.

From the wide spectrum of neurotrophic factors and cytokines, only a few draw attention of most researchers. Brain-derived neurotrophic factor (BDNF) is considered as one of the central players in pathogenesis of major depression since altered BDNF-dependent signaling in the brain of patients is suggested. Indeed, in postmortem brain samples of patients with major depression, BDNF receptor (TrkB) expression was reduced [10]. Moreover, in depressed subjects, downregulation of genes with high and intermediate BDNF dependency along with BDNF-independent genes was revealed in studies of postmortem brain. Interestingly, the changes were more expressed in men (potentially, because of low baseline expression in women). Reduced levels of the mature BDNF, but not its precursor pro-BDNF, were reported in sera, plasma, and platelets from depressed patients (see [11]). In addition to the decreased contents and expression of BDNF and its cognate TrkB receptor, the levels of nerve growth factor (NGF) and its receptor TrkA proteins and mRNAs are also lower in the hippocampus of suicide victims with major depression as compared to nonpsychiatric individuals [12]. However, we cannot exclude that other members of neurotrophin family may also be involved in pathogenesis of depression. Similarly, most of the studies in the field are focused on definite cytokines including interleukin- (IL-)1*β*, IL-6, and tumor necrosis factor-*α* (TNF-*α*) since the changes in circulating IL-1*β*, IL-6, and TNF-*α* were revealed in patients with major depression [2].

In this review, we will focus on data from experimental studies based on application of various stress procedures aimed to induce depression-like behaviors in rodents. Most of these data are correlative; however, data from transgenic models and studies on direct effects of NTFs and cytokines will also be present when appropriate.

## 2. Stress Conditions as a Basis for Depression Models

Brain responds to diverse challenges defined as either exteroceptive stress, involving cognitive processing of sensory information from the external environment, or interoceptive stress, detected through sensory neural or chemical cues from the internal environment [13]. Among clinicians, the term “stress” is generally taken as synonymous with psychological (exteroceptive) stress. Psychological or exteroceptive stressors fall into different categories, depending on nature, severity and chronicity of the stressor, the individual's gender and age during stress exposure, and the subjectively recognized threat. There is much evidence that excessive stress exposure to the brain, mediated through the neurotoxic effects of cortisol and neuroinflammation, induces damage to brain structure and function, impairing neuronal plasticity. This “glucocorticoid cascade hypothesis” may also be relevant to exploration of depression-related brain pathology since functional changes of HPA axis as well as alterations in brain structures, specifically hippocampus, have been consistently reported in major depression (see [14] for review). Exposure to stressors is one of the most popular approaches to model depression in laboratory animals (see [15–19]). Stressful events are considered to be one of the major predisposing factors for the development of mood disorders [20], and, although many important symptoms of depression (e.g., feeling of worthlessness, suicidal thinking) cannot be modeled in animals, this approach has high etiological (construct) and pharmacological (predictive) validities. Indeed, exposure to chronic stress or even to single stress episode may cause specific depression-like behavioral changes, which can be reversed by standard antidepressant treatments. Depression-like symptoms resembling core clinical symptoms, “depressed mood” and anhedonia, are assessed in rodent models using forced swim and tail suspension tests (immobility time) and the sucrose preference test (consumption of a 1%-2% sucrose solution) [21]. Stress-induced models of depression can be classified according to stressor properties (e.g., physical or social), duration (acute or chronic), and period (early life or adult) of its exposure. Although a single stress (e.g., forced swim stress) may induce depressive episodes, there are numerous clinical observations that the appearance of the disorder symptoms is usually related to chronic stressful life events (e.g., financial problems) [18]. Chronic paradigms like social defeat and chronic mild stress imply the exposure of rodents to natural stressors mimicking stressful events of everyday human life [19]. Frequently, the same stress paradigm models both anxiety and depression which have high comorbidity in humans.

Compelling evidence exists for existing sex differences in pathological conditions, including anxiety and depressive disorders with females more than twice as likely to be afflicted. However, most of the experimental studies on the depression-like consequences of stress exposure do not take into account this fact, and the studies are performed on male rodents. Therefore, most of the results reviewed here are from experiments in males excluding those specifically indicated. No doubt, general neglecting of gender difference in most experiments prevents correct interpretation of the results as well as successful translation of experimental data into clinic.

## 3. Interference into Early Life Development 

Adverse early-life experiences (e.g., childhood physical and even emotional neglect) have been implicated in later-on development of various psychiatric disorders, including depression [22, 23]. There is evidence that prenatal stress and childhood maltreatment are associated with the abnormally developing HPA system, as well as hippocampal volume reduction [4]. To understand how early environmental factors alter developmental processes resulting in psychopathology, a variety of animal models focused primarily on the effects of prenatal stress have been elaborated.

### 3.1. Prenatal Stress (PNS)

One of the most widely used models of PNS involves exposure of pregnant dams to restraint stress by placing them in restraint tubes for a separate period of time over several days. There are, however, apparent differences in reported responses which may be also explained by gender differences and different periods of PNS application. Thus, restraint stress performed daily during the last week of rat pregnancy significantly increased depression-like behavior in adult male, but not female offspring [24, 25]. In contrast, restraint stress given daily on gestational days 15–17 enhanced neonatal neurogenesis and differentiation processes of hippocampal neurons [26] as well as learning in adults [27]. Finally, another stressful procedure, a chronic unpredictable mild stress on gestational days 10–20, had no effect on depression-related behavioral measures in adult male offspring [28]. PNS was suggested to affect vulnerability or resilience to the onset of stress-related psychopathological conditions by influencing epigenetic mechanisms responsible for the onset of these phenotypes [29, 30].

BDNF plays a critical role during neuronal development and is able to modulate neuronal signaling in adult offspring of rat dams that were stressed during gestation. PNS has a negative impact on neuronal plasticity inducing a reduction of BDNF/Bdnf expression and an increase in the methylation of BDNF exon IV in adult rat brain, amygdala, and hippocampus, specifically [31–34]. PNS by prenatal dexamethasone treatment impaired activation of the BDNF exon IV to acoustic challenge in the PVN of adult male and female rats [35]. Yeh et al. [36] demonstrated that PNS transiently switched the direction of synaptic plasticity in hippocampal CA1 region, favoring long-term depression (LTD) and opposing the induction of long-term potentiation (LTP), and these PNS-induced changes in hippocampal plasticity were correlated with increasing endogenous pro-BDNF and decreasing of the mature form of BDNF (m-BDNF). PNS resulted in a significant decrease in the activity and expression of tissue plasminogen activator (tPA), a key serine protease involved in the extracellular conversion of pro-BDNF to m-BDNF. These results suggest that PNS downregulates tPA level in the hippocampus by inhibiting the conversion of pro-BDNF to m-BDNF. van den Hove et al. [30] showed that in newborn Fischer 344 rats PNS resulted in an approximately 50% decrease in brain cell proliferation just after birth in both genders with a concomitant increase in caspase-3-like activity accompanied by a decrease of BDNF protein content in the hippocampus.

PNS also induced a reduced BDNF expression in the prefrontal cortex and striatum of adult rats. Furthermore, when exposed to a chronic stress in adulthood, these rats displayed an altered regulation of BDNF expression in these brain structures, suggesting regional specificity of PNS effects [37]. The authors suggested that dysregulation of corticostriatal BDNF expression, along with respective changes in hippocampus, might contribute to permanent alterations in brain functions leading to increased susceptibility to psychiatric disorders.

Epigenetic changes in hippocampal neuroplasticity induced by PNS are critically sex-dependent and both the neurochemical changes and the behavioral outcome may diverge in males and females [38]. Moreover, they are strain-specific. Neeley et al. [39] studied Fischer, Sprague-Dawley, and Lewis rats and demonstrated multiple disparities in mRNA expression levels of BDNF and transcripts related to its processing and signaling in the three strains. Of the numerous splice variants transcribed from the BDNF gene, the transcript containing BDNF exon VI, was most aberrant in post-PNS animals. Protein levels of both uncleaved pro-BDNF and m-BDNF were also changed by PNS, but also strain-specific, as was intracellular signaling by phosphorylation of the neurotrophic tyrosine kinase receptor TrkB (NTRK2) and mitogen-activated protein kinase Erk1/2. Differential processing of BDNF after PNS in different rat strains demonstrates the importance of genetic background and has implications for human subjects where genetic differences may protect or exacerbate the effects of environmental stressors during fetal development.

PNS modifies neuroinflammation-related processes in adult brain. In C57BL/6 mice, PNS increased IL-1*β* mRNA level in the hippocampus, the total number of Iba1-immunoreactive microglial cells, and the proportion of microglial cells with large somas and retracted cellular processes [40]. It also modified responses to peripheral inflammation induced by systemic administration of bacterial lipopolysaccharide (LPS): LPS induced an increase in mRNA levels of IL-6, TNF-*α*, and IL-10 in the hippocampus of prenatally stressed mice but not of control nonstressed animals, as well as a higher proportion of Iba1-immunoreactive cells in the hippocampus with morphological characteristics of activated microglia in stressed animals. Conversely, adult offspring of dams that experienced nest material restriction (rather rear model of PNS) had decreased anti-inflammatory IL-10 in male, but not female, brains [41]. Similarly, only males showed increased expression of the innate immune recognition gene of toll-like receptor 4 (Tlr4) and its downstream effector, caspase-1.

### 3.2. Early Postnatal Stress (Handling, Maternal Separation and Deprivation, and Isolated Rearing)

Early handling involves exposure of neonatal rats to short periods of maternal absence (3–15 min) in a novel environment for the first 2-3 weeks of life. In adulthood, these animals display an attenuated response of the HPA axis to stress, reduced emotional arousal, and an increased glucocorticoid receptor (GR) expression in the hippocampus and prefrontal cortex [42, 43]. Based on these data, early handling has been suggested as an animal model of resilience to stress and stress-related psychopathology.

In contrast to neonatal handling, longer periods of maternal separation within the same 2-3 postnatal weeks result in an increased vulnerability to a depression-like syndrome. During separation sessions, pups are removed from mother for the periods of 3–8 h per day. Maternal separation for 180 min/day induced changes in the expression of tryptophan hydroxylase 2 mRNA in the dorsal raphe nucleus, one of the potential mechanisms through which adverse early life events lead to the increase in vulnerability to stress-related psychiatric disease [44]. Long-term effects of maternal separation depend on genetic factors [45]. Maternal deprivation paradigm involves a single separation event for 24 h. At different phases of development, this stressful exposure may lead to different outcomes. Faturi et al. [46] stated that the observed differences may be translated to humans, because, for example, before adolescence, there is a similar chance of developing depression or posttraumatic stress disorder (PTSD) after trauma, but after the age of 13, the risk for PTSD is higher [47]. Changes in maternal behavior were suggested as the key factor underlying long-term effects of early maternal separation for different duration on the offspring behavior [48]. This hypothesis was supported by a strong correlation between the levels of maternal care and the behavior of adult offspring [49, 50]. Though the exact mechanisms linking early environmental conditions and offspring behavior in adulthood through changes in maternal care remain unclear, these effects are accompanied by important changes in central levels of BDNF [49]. A large number of studies employ chronic postweaning social isolation as a rodent model of social deprivation. This model involves rearing rats individually during the developmental period from the day of weaning, which can range from PD 21 to PD 28 across studies, until the day of testing usually in late-adolescence or adulthood [51, 52]. Postweaning isolation rearing increased immobility in the FST in adolescence/early adulthood [53]. Environmental enrichment reversed some of the effects of postweaning social isolation [54].

Early maternal separation increased levels of neurotrophic factors (BDNF, NGF, and NT-3) in both the dorsal and ventral hippocampi [55]. Cerebellar mRNA and protein levels of BDNF and TrkB were significantly increased in mother deprived rats at PD16. However, by PD30, these parameters reached control levels. In contrast, the levels of mRNA and protein for NGF, TrkA, p75 NTR, and NgR (Nogo receptor) were unchanged at both ages examined [56]. The expression of mRNA for BDNF, TrkB, insulin-like growth factor-1 (IGF-1), and type 1 IGF receptor (IGF-1R) in Wistar rat pups separated from their mothers for 3 h per day during PD10 to 15 was enhanced on PD16 and 20 and then returned to baseline levels on PD30 [57]. Maternal separation (3 h per day from PD2 through 14) in Sprague-Dawley rat pups increased plasma corticosterone release and elevated NGF levels in the hippocampus [58]. Kikusui and Mori [59] revealed a higher HPA activity in mother separated pups to novelty stress. Neurochemically, the early-weaned male mice showed precocious myelination in the amygdala, increased corticosterone levels on PD14, decreased BDNF protein levels in the hippocampus and prefrontal cortex, and reduced BrdU immunoreactivity in the dentate gyrus. Marais et al. [60] separated the rat pups from their mothers for 3 h/day on PD2-14. This caused significant changes in levels of NGF and NT-3 in the dorsal and ventral hippocampus, increased basal corticosterone levels, and decreased ACTH levels in response to acute restraint stress. Separation from mothers downregulated neurotrophins in the ventral hippocampus, possibly as an effect of high corticosterone level, and increased neurotrophin levels in the dorsal hippocampus may reflect compensatory mechanisms against cell death [60]. The maternal separation caused a reduction in plasma ACTH levels but evoked hypersecretion of corticosterone when it was combined with stress in adulthood [61]. Removal of a mother from rat pups significantly decreased the number of BrdU-positive cells in the dentate gyrus, but treatment with fluoxetine restored the degree of proliferation [62]. Maternal separation of mouse pups from the dams at an early age increased the duration and augmented some of the symptoms of sickness behavior induced by proinflammatory cytokines [63].

Postnatal maternal separation in rats caused a reduction of GABAergic parvalbumin-containing interneurons in the prefrontal cortex in adolescence and that correlated with increased circulating levels of the proinflammatory cytokines IL-1*β* and IL-6 [64]. Maternal separation produced a significant downregulation of the expression of six cytokine genes: chemokine ligand 7, chemokine receptor 4, IL-10, IL-1*β*, IL-5 receptor, and integrin *α* M. These cytokines may mediate the effects of early adversity on subsequent immunosuppression [65]. Carboni et al. [66] subjected to maternal separation the genetically selected rats (Flinders Sensitive Line, FSL) and made comparisons of corticosterone, cytokines, BDNF, and C-reactive protein levels with those of the FRL controls. Significant increases were detected in leptin, IL-1*α*, and BDNF, while C-reactive protein was significantly reduced.

Thus, early life stress of different kind affects systems of both neurotrophic factors and cytokines in rodent brain, and these disturbances are suggested to be involved in depressive-like behavior of adult animals.

## 4. Stress Exposure during Adulthood

### 4.1. Social Isolation

Although social isolation was suggested to be of particular relevance for juvenile/adolescent rats and mice, this paradigm is also used for adult rats [18, 52]. Compared with other stress-induced models, this model has received less attention due to missing data. For example, in one study, only 2 months of social isolation were enough for producing the effects useful as behavioral model of depression [67], though some other studies could not reproduce this effect. Prolonged social isolation of adult rats reduced sucrose drinking [18]. Animals subjected to social isolation avoided the central zone in the open field test and spent less time swimming with a longer immobility in the FST. The stressed animals also exhibited a marked hypertrophy of the adrenal gland cortex accompanied by a decrease in the serum corticosterone level [68]. The increase in synaptosomal polysialic neural cell adhesion molecule (PSA-NCAM), a molecular plasticity marker in the hippocampus of chronically isolated rats, was also observed, while subsequent treatment with fluoxetine brought it back to the control level [68]. Liu et al. [69] studied depression-like behaviors of single- and group-housed mice in the elevated plus-maze, open field, and FST after repeated restraint stress. Chronic restraint stress significantly decreased time in the open arm of the elevated plus-maze and increased immobility time in the FST in single-housed mice with no effects on the behavior in group-housed mice. Chronic stress upregulated levels of serum corticosterone and reduced the hippocampal GR in single-housed animals, but not in group-housed mice [69]. Twelve hours of social isolation produced depressive-like behavior in mice, enhanced corticosterone levels, and reinstated retrieval of a forgotten discriminative aversive (i.e., negatively valenced) task [70]. Depressive-like behavior was typical for social isolation since 12 h crowding neither induced such behavior nor enhanced retrieval, although it increased corticosterone levels similarly to social isolation. A 6 h period of social isolation immediately after contextual fear conditioning impaired memory for context fear measured 48 h later and decreased BDNF mRNA in the dentate gyrus and the CA3 region of the hippocampus assessed immediately after the isolation [71]. Social isolation for 1 or 3 h after contextual fear conditioning also increased IL-1*β* protein in the hippocampus and cerebral cortex [72]. The isolated mice exposed to chronic mild stress showed higher basal corticosterone and lower IL-2 and IL-4 as well as splenocytes proliferation compared to group-housed male mice [73]. Social isolation increased versus anti-inflammatory cytokine balance and altered kynurenine metabolism with a decrease in neuroprotective ratio in rats [74].

### 4.2. Learned Helplessness and Chronic Mild Stress Models of Depression

The learned helplessness (LH) paradigm implies subjecting rodents to uncontrollable and inescapable aversive stimuli like electric foot shock, tail shock, or loud acoustic sounds [75]. Following this exposure, animals develop a state of “helplessness” evidenced by the absence of escaping motivation during the reexposed sessions with an easy escape route [18]. Rodents subjected to inescapable tail shock in addition to impairment of escape behavior showed weight loss, agitated locomotor activity, sleep disturbances, decreased libido, deficit in cognitive behavior, and increased corticosterone levels [76]. Pharmacological treatment with antidepressants can reduce these behavioral changes [77]. The chronic mild stress (CMS) paradigm involves the exposure of adult rats and mice to a variety of relatively mild unpredictable stressors in a random order over several weeks [15, 18, 19, 52, 75, 78]. The stressors include isolation housing and grouping, overnight and intermittent illumination, cage tilting, food and water deprivation, cold stress (4°C), white noise, restraint, forced swimming. As a result, the animals exhibit the long-term behavioral, neurochemical, neuroimmune, and neuroendocrine alterations, including depressed mood, anhedonia, and sleep disturbances, resembling those observed in depressed patients. The alterations induced by long-term stress can be reversed by chronic, but not acute, treatment with antidepressant medications [79]. It should be noted that the CMS model was shown to be sensitive to subtle variations in design and, thus, has a poor interlaboratory reliability, particularly in rat studies [75].

Involvement of cytokines in an LH model of depression is poorly studied. LH elevated inflammatory T helper 17 (Th17) T cells in the mouse brain [78] indicating that depression-like behavior in this paradigm may be related to activation of inflammatory mechanisms. Moreover, IL-6 knock-out mice exhibited resistance to LH [80].

In CMS, animal stress triggered the production of inflammatory cytokines, such as IL-1 and IL-6 [81–83]. Immobilization stress alone increased IL-1 mRNA expression in the hypothalamus [84]. It was shown that inescapable shock increased brain IL-1*β* in adrenalectomized rats 2 h after stress [85]. Mild inescapable foot shock significantly increased production of IL-1*β* and TNF-*α* by isolated alveolar macrophages [86]. Male Sprague-Dawley rats exposed for 4 weeks to CMS demonstrated a reduction of sucrose intake without any effect on water intake [87]. Humoral assays showed the increased plasma levels of TNF-*α*, IL-1*β*, plasma renin activity, aldosterone, and corticosterone in the CMS-exposed rats. Moreover, brain cytokine concentrations negatively correlated with sucrose consumption suggesting that higher levels of cytokines were responsible for more expressed anhedonia. After 3-week-long CMS, C57BL/6 mice demonstrated the decreased thymus weight and increased production of IL-1 [88]. Goshen et al. [89] showed that mice subjected to CMS for 5 weeks exhibited depressive-like symptoms, including decreased sucrose preference, reduced social exploration and adrenocortical activation, and increased IL-1*β* level in the hippocampus. In contrast, mice with deletion of the IL-1 receptor type I (IL-1rKO) or mice with transgenic, brain-restricted overexpression of IL-1 receptor antagonist failed to display CMS-induced behavioral or neuroendocrine changes. Similarly, whereas in wild-type mice CMS significantly reduced hippocampal neurogenesis, no effect was observed in IL-1r KO mice. The sucrose intake was significantly decreased, while corticotropin-releasing factor, cortisol, IL-6, and TNF-*α* levels increased in CMS-treated Sprague-Dawley rats compared to controls [90]. Xiu et al. [91] reported that CMS increased serum TNF-*α* production and serum concentration of IL-6 both in tumor-bearing and non-tumor-bearing rats, although Grippo et al. [87] could not find changes of IL-6 in brain and serum of CMS-treated animals. In contrast to this, Chourbaji et al. [80] found that IL-6 (−/−) mice showed resistance to stress-induced helplessness. This resistance could occur due to a lack of IL-6, since stress increased IL-6 expression in hippocampus of wild-type animals. In rats exposed to CMS, a high expression of proinflammatory cytokines IL-1*β*, TNF-*α*, and IL-6 and low expression of anti-inflammatory cytokines TGF*β* and IL-10 were demonstrated; thus, higher ratios of TNF-*α*/IL-10 and IL-6/IL-10 were evident in the brain [92]. Simultaneously, BDNF mRNA decreased significantly in the hippocampus and hypothalamus of rats subjected to CMS.

In Swiss albino mice, CMS impaired memory in object recognition test and object location test was accompanied by the increased plasma levels of IL-1*β*, IL-6, and TNF-*α*, as well as the enhanced plasma levels of corticosterone, corticotrophin-releasing hormone, and ACTH [93]. In addition, severe neuronal cell damage was found, while BrdU-positive cells and the expression of BDNF in the dentate gyrus of the hippocampus were decreased after 5 weeks of CMS procedure. Liu et al. [94] found that CMS-related depression-like behavior in rats was accompanied by the following changes: increased serum corticosterone level, decreased 5-HT level, increased IFN-*γ* and TNF-*α* levels, and elevated indoleamine 2,3-dioxygenase (IDO) activity in prefrontal cortex. Moreover, the level of 5-HT inversely correlated with the IDO level. Regular swimming exercise ameliorated depressive symptoms induced by CMS, corticosterone levels, and the respective neurochemical changes.

Rats trained in the LH paradigm showed significantly higher serotonin turnover in the orbitofrontal cortex and lower levels of BDNF in the hippocampus than control animals [95]. This effect was also observed in young and old rats bred for LH [96]. Treatment with lamotrigine prevented downregulation of BDNF expression in the frontal cortex and hippocampus in LH [97]. Decreased BDNF and CREB mRNAs in the hippocampus after LH correlated with increased corticosterone in blood plasma [98]. A single bilateral infusion of BDNF into the dentate gyrus of hippocampus produced an antidepressant effect in both the LH and FST that was comparable in magnitude with repeated systemic administration of a chemical antidepressant [99]. However, Schulte-Herbrüggen et al. [100] did not observe any changes in the BDNF contents in the hippocampus and frontal cortex, and Greenwood at el. [101] reported that LH behaviors are independent of the presence or absence of hippocampal BDNF because blocking inescapable stress-induced BDNF suppression did not always prevent LH and LH did not always occur in the presence of reduced BDNF. Along with BDNF, NGF and ciliary neurotrophic factor (CNTF) may be related to LH. Indeed, NGF content was elevated 6 h after training of rats in the LH paradigm [100], and CNTF knock-out mice were more prone to depression-like behavior in the LH model [102].

CMS produced cognitive deficits in rats tested in Morris water maze and novel object recognition task, elevation of serum corticosterone and decrease of BDNF levels in the prefrontal cortex and hippocampus, along with decreased phosphorylation of extracellular signal-regulated kinase (pERK) and cAMP response element-binding protein (pCREB) [69]. CMS significantly affected the survival of new-born cells in the granule cell layer but did not influence their proliferation or differentiation [62]. No changes in BDNF mRNA levels in the dentate gyrus could be observed. The BDNF levels did not change after 5 weeks of restraint stress either [103]. The lack of CMS-induced changes in expression of BDNF mRNA in the hippocampus and amygdala was demonstrated by Allaman et al. [104] and Lucca et al. [105]. Thus, some authors claim that CMS may reduce the survival of new-born cells without any significant effects on neurogenesis. However, in other studies, a severe impairment of hippocampal neurogenesis induced by CMS was demonstrated [106, 107]. Jiang et al. [108] showed that CMS substantially decreased neurogenesis and dendritic spine density in mice. The average BDNF mRNA expression in their experiments was decreased in the hippocampus of mice exposed to CMS as compared to unstressed control. The expression of pERK1/2 (the active form of ERK1/2) in the hippocampus was much lower in CMS-treated rats relative to controls, while the levels of ERK1/2 remained unchanged in all groups; pCREB level was significantly lower in the hippocampus of CMS mice. Similarly, CMS reduced BDNF expression and inhibited phosphorylation of CREB (Ser-133) in the dentate gyrus, whereas no significant effects were observed in the other parts of hippocampus [109]. CMS significantly decreased cytogenesis (measured by BrdU) in the ventral part of the hippocampal formation [110]. Rats treated with antidepressants showed recovery of neurogenesis. First et al. [111] showed that CMS reduced BDNF levels in male Sprague-Dawley rats, while the selective norepinephrine reuptake inhibitor, reboxetine, reversed this effect of CMS and increased BDNF receptor (TrkB) levels. Reboxetine elevated hippocampal ERK phosphorylation in both stressed and unstressed rats. Vithlani et al. [112] recently demonstrated that abilities of BDNF to modify neurogenesis and depressive-like behaviors depended on phosphorylation of tyrosine residues 365/367 in the GABA- (A-) receptor *γ*2 subunit.

Vascular endothelial growth factor (VEGF) is an important trophic factor associated with active sites of neurogenesis and formed by proliferative cells that present an endothelial phenotype in 37% of the cases [113]. VEGF expression was reduced in hippocampal dentate gyrus in a CMS model [114] although the other authors could not find changes of VEGF associated with animal models of stress [115]. Some antidepressants upregulated VEGF expression while the local administration of this trophic factor produced an increase in hippocampal proliferation [116]. In addition, silencing of hippocampal VEGF [117] or use of its receptor antagonists Flk-1 blocked its antidepressant-like effect and decreased expression of doublecortin (DCX), a marker of newborn neurons [115]. CMS also significantly decreased the level of NGF in the frontal cortex of the animals [118].

### 4.3. Social (Resident-Intruder) Defeat Model

The social defeat stress model involves daily introduction of a physically superior aggressive animal into the home cage of a resident animal for a period of several weeks [119]. Chronically defeated rats showed behavioral changes, including decreased motility and exploratory activity, increased immobility in the FST, and reduced preference for sweet sucrose solution (anhedonia) [120, 121]. Defeated animals also exhibit reduced social interaction (social avoidance) and increased anxiety-like behavior. Chronic antidepressant treatment has been demonstrated to reverse the social deficits induced in chronically defeated animals [122]. Relation of this model to depression is suggested, for example, from the observation of social avoidance in humans suffering from depression [123]. At the same time, social defeat model may also relate to panic disorder, social phobia, or PTSD [52, 119]. In a social defeat model, the loser rats exhibit increased ACTH, enhanced corticosterone, and decreased testosterone levels compared to controls [124].

Patki et al. [125], using a resident-intruder (social defeat) model of social stress, observed a significant decrease in the body weight and long-term memory impairments in the socially defeated rats compared to controls. Significant increases in ERK1/2 and IL-6 levels and decreases in calcium/calmodulin-dependent protein kinase type (CAMK) IV, CREB, and BDNF were demonstrated in the hippocampus of socially defeated rats, but not in the prefrontal cortex and amygdala. Analysis of cortical homogenates of rats subjected to dominant-submissive relationships competing for a food reward revealed the elevated levels of IL-6 but not IL-1*β*; however, there were no significant increases in IL-6 or IL-1*β* in the cortex of submissive animals relative to dominant subjects [126]. Yet, in competing pairs, in which the hierarchy was unstable and rats continued to fight for dominance, both subjects of the pair demonstrated significant elevations of IL-6 (but not IL-1*β*) in the cortex relative to pairs, in which dominance level was stable [126]. Gómez-Lázaro et al. [124] studied behavioral profiles in 6-week-old male mice in response to chronic social defeat stress for 21 consecutive days. On the basis of confrontation on day 21, the mice were divided into two groups: active and passive. Passive mice had a high level of immobility, low nonsocial exploration, and higher plasma corticosterone concentrations as compared to active mice. Three days after the last defeat, passive mice had lower corticosterone levels, higher levels of IL-6 and TNF-*α* in the spleen, and lower hippocampal BDNF levels than active and manipulated-control mice. The only differences observed in active mice in relation to the manipulated control were higher plasma corticosterone (day 21) and TNF-*α* levels [124]. In the absence of a prior stressor experience, the social defeat challenge did not affect prefrontal IL-1*β* or TNF-*α* mRNA expression but increased the expression of IL-6 [127]. In mice that had initially been repeatedly defeated, IL-1*β* and TNF-*α* expression was enhanced after the social defeat challenge. In contrast, the increase in IL-6 expression in initial social defeat stressor was limited to subsequent challenge with social defeat. Previous social stressor experience also limited the corticosterone increase ordinarily elicited by social defeat [127]. Wu et al. [128] in social defeat model in mice demonstrated impaired expressions of glucocorticoid receptors mRNA and BDNF mRNA in the hippocampus and increased level of corticotrophin-releasing hormone mRNA in hypothalamus, as well as increased levels of IL-6 and TNF-*α* in serum. Fiore et al. [129] found that dominant animals had higher levels of BDNF mRNA in the subventricular zone and hippocampus than did subordinate animals. Conversely, subordinate animals exhibited higher levels of NGF compared with dominant animals in these neurogenic regions. BDNF mRNA in mice exposed to a 10-min social defeat was lower than that in nondefeated animals [130]. Berton et al. [131] demonstrated upregulation of BDNF protein in the nucleus accumbens following 10 days of chronic social defeat. Mice with overexpressed, dominant negative truncated splice variant of the BDNF receptor TrkB (TrkB.T1 mice) exhibited smaller changes in body weight and food intake and had more consistent and long-lasting social avoidance than their wild-type counterparts after social defeat [132]. Using the same (a resident-intruder) model, Taylor et al. [133] showed that losing animals had significantly more BDNF mRNA in the basolateral and medial nuclei of the amygdala as compared to winning animals and controls. Winning animals had significantly more BDNF mRNA in the dentate gyrus of the dorsal hippocampus than did losing animals and controls. The level of tegmental BDNF depended on the duration of defeat: in episodically defeated rats BDNF was increased, whereas in the continuously subordinate rats the level of BDNF decreased [134]. Defeat was accompanied by elevated levels of serum corticosterone and NGF [135]. Repeated exposure to an intruder induced a state of glucocorticoid resistance in peripheral immune cells. Glucocorticoid resistance developed in animals that exhibited a subordinate behavioral profile, combining a low tendency for social exploration and a high level of submissive behavior in response to the intruder's attacks. However, glucocorticoid resistance was also linked to the presence of injuries due to fighting but not to changes in systemic levels of either corticosterone or NGF [135]. Fibroblast growth factor, FGF, was significantly downregulated following social defeat; specifically, FGF2 and FGFR1 mRNA expression was decreased in various subfields of the hippocampus [136].

Mice adrenalectomized before social defeat showed enhanced behavioral resilience and increased survival of adult-born hippocampal neurons compared with sham-operated defeated mice [137]. However, mice lacking hippocampal neurogenesis did not show protective effects of adrenalectomy. van Bokhoven et al. [138] studied the effects of repeated social defeat and subsequent individual housing for 3 months on adult hippocampal neurogenesis (a process highly dependent on NTFs balance) in rats. In social defeated rats, the total DCX(+) cell number was significantly reduced mostly for older DCX(+) cells with long apical dendrites, whereas younger cells remained unaffected. There was a significant decrease in cell proliferation in mice that received 10 social defeats [139]. This decrease was correlated with the intensity of the defeat experiences. Cell proliferation was only slightly inhibited after a single defeat and this effect was not significant. Three defeats within a 5 h period had no effect on levels of proliferation. Offensive aggressive stress in the residents did not result in any changes in hippocampal cell proliferation [139].

### 4.4. Forced Swim and Restraint Models of Depression

FST and tail suspension test (TST, “dry” version of the FST) are the most widely used tests for the preclinical screening of antidepressants; however, they also provide models to study the neurobiological mechanisms underlying depression development and therapy [75]. In the classical FST elaborated by Porsolt et al. [140, 141] on rodents, the animals are placed twice for 15 min and 5 min in an inescapable cylinder of water in a 24 h interval, and amount of their immobility time is measured. Compared with the first session, during the second swim, animals demonstrate an increase in duration of immobility that is interpreted as a state of “behavioral despair,” which is attenuated by subacute [75] and chronic administration of antidepressant drugs [142, 143]. Swim stress procedure can be performed once, that is, acute swim stress, or repeatedly over several days or even weeks, that is, chronic swim stress. The restraint stress paradigm consists in daily enclosing rodents in narrow tubes or cages, which restrict their movement for a period of 15 min to 6 h a day. Exposure of adult rats to restraint stress for 2.5 h a day for 13 consecutive days promoted a significant increase in immobility during the FST. The increase in immobility observed in stressed animals was returned to control values by chronic treatment with antidepressant sertraline [144]. Recently, Koike et al. [145] demonstrated that the BDNF/TrkB signaling pathways may be involved in antidepressant-like effects of a group II metabotropic glutamate receptor antagonist on tail suspension and the novelty-suppressed feeding models of depression. Restraint stress (2 h a day for 14 days) in rats produced increases in the serum corticosterone level and the expression of corticotropin releasing factor in the hypothalamus as well as decrease in neuronal tyrosine hydroxylase immunoreactivity in the ventral tegmental area and the expression of BDNF mRNA in the hippocampus [146]. The rats subjected to restraint stress revealed increased duration of immobility in the FST and decreased sucrose consumption. TNF-*α* receptor 1 (TNFR1) knockout mice exhibited an antidepressant-like behavior in the FST and in the TST as compared with the wild type mice [147].

### 4.5. Olfactory Bulbectomy Model of Depression

Bilateral removal of the olfactory bulbs (OB) in rats results in behavioral, neurochemical, neuroendocrine, and immune alterations similar to those seen in patients with major depression. OB-induced changes are reduced by chronic but not acute antidepressant treatment as also observed in depressed patients. In response to environmental stress, OB rats show increased exploratory activity, psychomotor agitation, decreased libido, eating disorder, and deficit in long-term memory [148]. These animals also reveal an increased immobility time in the FST, hyperactivity in an open field arena, and anhedonic response in sucrose preference test [149]. Additionally, OB removal produces ventricular enlargement and decrease of cortical, hippocampal, caudate, and amygdalar volumes [150]; decrease of protein expression of NMDA receptor subunit NR1 (but not NR2A, B) in prefrontal cortex, hippocampus, and amygdala; and decrease in phosphorylation of CREB in the prefrontal cortex and hippocampus [148]. Such behavioral, neurotransmitter, and structural changes are related to neuroinflammatory events in OB rats. Song et al. [151] found an increased expression of corticotropin releasing factor in the hypothalamus and increased secretion of corticosterone in OB rats as compared to sham-operated controls. Rinwa et al. [149] revealed increased levels of inflammatory cytokines (TNF-*α*) and caspase-3 along with a marked reduction in BDNF in the brain of OB rats. In OB rats, NGF mRNA expression was substantially lower in the hippocampus while levels of IL-1*β* and prostaglandin E2 increased in the serum and brain [151]. The anti-inflammatory drug celecoxib significantly reduced blood prostaglandin E2, IL-1*β*, and corticosterone concentrations, increased NGF expression, and normalized behavior in OB rats. Recently, Freitas et al. [152] showed that OB removal in mice caused significant increases in ERK1 and CREB phosphorylation as well as in the expression of BDNF; all these effects of OB could be prevented by fluoxetine administration.

Though some data are contradictory, there is no doubt that brain systems of neurotrophic factors and cytokines are principally involved in stress-induced depressive-like behavior in adult rodents. The definite changes are related to the specific details of stress (modality, duration, and severity) and animals used (species, strain, and gender).

## 5. Cytokines and Depression Models

The data related to NTFs and cytokine changes in rodents subjected to exteroceptive stress of different nature and reviewed in Chapters 3-4 still seem fragmentary and some of them quite contradictory. Obviously, the puzzle game illustrating the stress-related pattern of NTFs and cytokines in rodent brain is far away from being solved yet. In this section, the data related to interoceptive stress-induced depressive behavior will be presented. It should be noted that the interest in studies in this field is growing rapidly during the last five years.

It has been hypothesized that cytokines may cause depressive illness in man. Dunn et al. [153] have reported that several groups of observations may support this hypothesis. First of all, treatment of patients with cytokines can produce symptoms of depression. Second, activation of the immune system is observed in many depressed patients. Third, depression occurs more frequently in those with medical disorders associated with immune dysfunction. These data were additionally supported by experimental evidence on induction of sickness behavior, which resembles depression in animals treated with bacterial LPS or IL-1 in order to activate the immune system, and chronic treatment with antidepressants has been shown to inhibit sickness behavior induced by LPS. Several cytokines can activate the HPA axis, which is frequently activated in depressed patients. Additionally, some cytokines activate cerebral noradrenergic and serotonergic systems, another common symptom observed in depressed patients and implicated in major depressive illness and its treatment.

### 5.1. Cytokines as Inducers of Sickness Behavior

Sickness behavior is one of the most studied effects of cytokines on the brain. This model may provide insight into the etiology and the mechanisms underlying some symptoms of major depressive disorder. Sickness behavior is a well-coordinated complex of subjective, behavioral, and physiological modifications accompanying progression of infectious diseases and may be considered as a form of adaptive response to infection [154]. These modifications are considered as a consequence of the central effects of cytokines, which are synthesized by cells of the immune system on periphery and transported into the CNS by active transport via the blood-brain barrier. However, induction of sickness behavior by LPS injection resulted in an increase in local expression of IL-1*β*, IL-6, and TNF-*α* mRNAs in the hypothalamus [155]. This was followed by appearance of respective proteins influencing behavioral manifestations of sickness in animals. These events were accompanied by activation of microglia and astrocytes in the dentate gyrus [156]. IL-1 receptor antagonist (IL-1ra) and anti-TNF-*α* antibody attenuated the signs of sickness behavior improving social interaction between the animals [154, 157]. In contrast to proinflammatory cytokines, the effects of intracerebroventricular administration of anti-inflammatory cytokines are controversial. Thus, anti-inflammatory IL-13 aggravated [158] whereas IL-10 ameliorated behavioral indices of sickness behavior [159] induced by peripheral LPS administration in rats. The effects of another anti-inflammatory cytokine IL-4 were also dual and depended on dose and time of its administration. Simultaneous injections of LPS and IL-4 significantly increased whereas IL-4 administration 12 h prior to LPS completely blocked the indices of sickness behavior [160]. In spite of some similarity between sickness behaviors and depression symptoms, they are not identical and each has distinct features. Therefore, the value of sickness behavior as an animal model of major depressive disorder is limited, and extrapolating results from the model to the human disorder should be performed with caution [153].

### 5.2. Effects of Cytokines in Conventional Models of Depression-Like Behavior

Sickness behavior is usually studied using social interaction or social exploration models. Other behavioral features, which may be depressed after systemic LPS administration, are general activity, feeding, nest-building. In addition, there are several tests widely used (FST and TST, see Section 4.4) for assessment of the effects of antidepressants and considered to some extent as behavioral models of depression-like behavior in humans. It has been shown that intraperitoneal administration of LPS increased immobility in the FST and this effect prevented by minocycline administration [161, 162]. LPS-treated animals demonstrated depression-like behavior in the FST decreasing climbing and increasing floating when sickness behavior had abolished, that is, 24 h post-LPS [163]. LPS administration also affected sickness-associated behaviors to a different extent in male and female rats, as assessed in the FST, the hot plate test and the open-field arena. LPS-treated female rats coped better with the stressful FST procedure, as evidenced by an increase in swimming duration. The effects of LPS treatment appeared to be more robust in male rats, as far as suppression of locomotor activity is concerned, while the antinociceptive properties of LPS were evident in both sexes though showing sex-dependent kinetics. Moreover, when traditional measures of sickness (i.e., sucrose consumption, social exploration, food intake) were assessed, males and females appeared to be similarly affected, except for food intake [164]. Two h after LPS treatment, increased indices of depression-like behavior including immobility in the TST and FST, decreased locomotor activity and total number of transitions between the light and dark compartments in a light/dark chamber were demonstrated [165]. Twenty-four h after LPS administration, the increased immobility time in the TST and FST without any effect on spontaneous locomotor activity was observed [166]. These depressive-like behavioral signs were associated with elevated TNF*α* and IL-6 mRNA expression and prevented by fluoxetine treatment. The LPS-treated mice exhibited depression-like behaviors and significantly increased levels of pro-inflammatory cytokine IL-1*β* protein and NLRP3 inflammasome mRNAs [167].

The effects of systemic LPS administration are probably mediated via Toll-like receptors (Tlr). Interestingly, LPS treatment enhanced behavioral despair in the FST induced by chronic mild stress [168, 169] and this effect of LPS was due to its interaction with Tlr-4, which is upregulated in response to stress. This may explain why stress may aggravate depression-like symptoms usually associated with diseases. LPS-induced depression was associated with increments in IL-1*β* content in plasma and prefrontal cortex, TNF-*α* in plasma, and decreased nitrergic neurotransmission evident in the striatum and prefrontal cortex [170]. These effects could be ameliorated by treatment with classical antidepressants such as imipramine [169]. Depressive symptoms are known to correlate with alterations of the aldosterone system. Mineralocorticoid aldosterone is involved in the regulation of inflammation and increases LPS-induced IL-1*β* mRNA expression in the prefrontal cortex and cerebrospinal fluid [171]. Cotreatment of rats with aldosterone and LPS resulted in more expressed depression-like symptoms.

Intracerebroventricular (icv) administration of LPS at a dose of 100 ng also increased duration of immobility in the FST. The effects were associated with elevated steady-state transcripts of TNF-*α*, IL-6, and the inducible isoform of nitric oxide synthase (iNOS) in the hippocampus in the absence of any change in IFN*γ* mRNA [172]. Similarly, 10 ng of LPS, injected into the brain of mice, increased the time of immobility in the TST [173]. Pretreatment with IGF-I or antidepressants significantly decreased duration of immobility in the TST in both the absence and the presence of LPS. Park et al. [173] have measured steady-state mRNA expression of inflammatory mediators in the whole brain using real-time RT-PCR and demonstrated that LPS increased, whereas IGF-I decreased, expression of inflammatory markers including IL-1*β*, TNF*α*, iNOS, and glial fibrillary acidic protein (GFAP). Moreover, IGF-I increased expression of BDNF. Depression-like behavioral symptoms induced by icv LPS injection (immobility in the FST but not sickness) were less expressed in mice with genetic knockout of IL-1*β* converting enzyme (caspase-1), which converts pro-IL-1*β* into active mature IL-1*β*, additionally indicating involvement of central cytokines in pathogenesis of depression [174]. Studies on the delayed effects of icv LPS or individual cytokine treatment are quite rare. We have reported that approximately 3 months after icv injection of human TNF-*α*, there were not any effects on immobility duration although the initial struggling time was significantly shorter in TNF-*α* compared to vehicle-treated rats [175].

Anhedonia, anorexia, body weight loss, and reduced locomotor, exploratory, and social behaviors are important components of the depression-like syndrome induced by immune activation with various acute and chronic immune challenges in rodents [176–180]. Chronic treatment with antidepressants (imipramine or fluoxetine) attenuates many of the behavioral effects of LPS, as well as LPS-induced changes in body temperature, adrenocortical activation, hypothalamic serotonin release, and the expression of splenic TNF-*α* mRNA. Interestingly, serotonin transmission may be involved in LPS-induced anhedonia because this effect was completely abolished in male serotonin transporter knockout rats [181].

Repeated treatment with LPS may result in tolerance development including the anhedonic effect [182]. An attenuation of anhedonia was observed after pretreatment with minocycline, an anti-inflammatory agent [178]. However, intermittent protocol of LPS treatment such as repeated injections once daily for 5 days in increasing doses for the first three days, which were then gradually decreased on days 4 and 5, at a one-month interval for 4 consecutive months induced chronic anhedonia (estimated by the preference to drink 1% sucrose) lasting for at least 7 weeks [183]. Chronic LPS administration significantly decreased thymus weight, proliferative activity of splenocytes, production of IFN*γ* and IL-10, as well as increased superoxide and corticosterone production. Treatment with fluoxetine for 3 weeks abolished the effects of LPS.

Individually housed mice responded to LPS with increased depressive-like behavior as compared to group-housed mice [184]. The duration of this effect on FST behavior depended on the genetic background. The behavioral changes induced by LPS in C57BL/6 mice were associated with a particularly pronounced rise of IL-6 in blood plasma within 1 day posttreatment and with changes in the time course of the corticosterone response to the FST. Central administration of recombinant mouse IL-6 produced depressive-like phenotypes in mice, which were not accompanied by IL-1*β*-induced increases in the brain tissue or IL-1*β*-related sickness behavior typical of a general CNS inflammatory response [126]; however, these behavioral manifestations were resistant to current classes of antidepressant medications. The importance of cytokines, specifically IL-1*β*, for the expression of depression-like phenotype in animals was additionally supported by the data demonstrating that chronic IL-1*β* expression in rat brain by adenoviral-mediated gene transfer resulted in prolonged depression of spontaneous behavior associated with chronic leukocyte recruitment and axonal injury [185]. Blockade of IL-1*β* receptor (IL-1R) by either an inhibitor or IL-1R null mice prevented the antineurogenic effect of stress and blocked the anhedonic behavior caused by chronic stress exposure [186]. Brain-directed overexpression of human soluble IL-1 receptor antagonist, hsIL-1ra, resulted in increased locomotion and decreased habituation, an anxiolytic effect, but did not influence motor performance [187]. However, long-term local expression of IL-1 in the hippocampus, reflecting local inflammation in this specific brain region, was not accompanied by substantial impairments in anxiety or locomotor activity [188]. Early and adult hippocampal overexpression of anti-inflammatory cytokine TGF-1*β* had opposite effects on depression-like behavior: adult TGF-1*β* overexpression decreased immobility in both TST and FST, whereas early hippocampal overexpression of this cytokine increased depression-like behavior and decreased social interaction in mice [189].

### 5.3. Effects of Cytokine Treatment on Depression-Like Behavior: Prenatal, Early Postnatal, and Pubertal Periods

Prenatal and early postnatal development is a vulnerable time of ontogeny, during which wiring of the CNS is fine-tuned and receptive to changes in environmental conditions. In rodents, early-life stressors introduced during the first week of life have been demonstrated to have long-term, permanent consequences on behavior and physiology. It has been demonstrated that rats exposed to low-dose of LPS, a potent activator of the HPA axis, on embryonic day 10.5 in order to mimic mild maternal infection exhibited more depression-like behaviors and had reduced adult neurogenesis and BDNF. Functions of dopaminergic and serotonergic neurons were also reduced in the offspring. The behavioral abnormalities and reduction in adult neurogenesis could be reversed by chronic fluoxetine treatment [190]. However, prenatal exposure to LPS on gestational day 17 did not result in expression of depression-like symptoms in adult C57/Bl6 offspring [191].

Early life immune challenge with LPS on PD 3 and 5 modified CNS serotonergic-related gene expression during postnatal development [192]. Early LPS challenge also resulted in a transient decrease in corticotropin releasing hormone mRNA expression in the CA1 and CA3 regions of the hippocampus accompanied by increased hippocampal GR mRNA expression in the CA1 region between PD14 and PD21. This was followed by increased hypothalamic corticotropin releasing hormone expression in LPS-mice on PD28 [193]. It has been demonstrated that immune challenge with LPS on PD 3 and 5 resulted in long-term alterations to adult stress-related behaviors. One of the most consistent observations is that exposure to LPS in early life induces increased anxiety-like behavior in adulthood. LPS-treated animals spent more time in the closed arms, exhibited fewer entries to the open arms of an elevated plus-maze, reduced exploratory behavior in the holeboard apparatus, and increased risk assessment behavior in the open field apparatus [194–197]. On the other hand, Tenk et al. [198] have reported decreased anxiety-like behavior on the light-dark test in female but not male adult rats; however, both males and females exhibited situation anxiety-like behavior in the hyponeophagia test. Early pubertal treatment with LPS may enhance depression-like symptoms in ovariectomized female mice and this effect was strikingly increased after estradiol administration [199]. The effect of LPS neonatal treatment at PD14 was not evident for sucrose preference or for total fluid intake and these data may be suggestive of dissociation between inflammation and anhedonic behavior [200].

In spite of various specific models and approaches, the vigorous use of cytokines for modeling depressive-like behavior is consistent with their intimate involvement in these behavioral disturbances.

## 6. Conclusion and Perspectives

**Figure 1:**
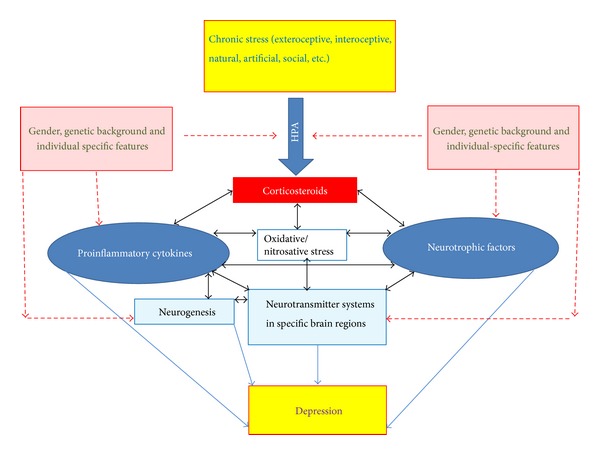
The stress hormones, neurotrophic factors, and cytokines are implicated in the complex network of molecular and cellular processes affecting brain function and important for depression pathogenesis. Oxidative/nitrosative stress is tightly involved in many mechanisms affecting the balance of neurotrophic factors and cytokines. Disturbances of neurogenesis in the subgranular zone as well as alterations in neurotransmitters and their receptors in specific brain areas directly contribute to depression-like behavior. All these systems and mechanisms are also dependent on genetic background and gender.

The stress hormones, NTFs, and cytokines are involved in the complex network of molecular and cellular processes affecting brain function and important for depression pathogenesis (Figure 1). The main alterations of NTFs and cytokines in different rodent models of depression induced by stress of different kind are summarized in Table 1. One apt way of partitioning stress models is to consider two basic classes, interoceptive (systemic) and exteroceptive (neurogenic), a classification based on the similarities in the overall patterns of activation responses seen as a consequence of exposure to a range of perturbations in the internal versus external environments [13]. Though the response to stressors of each class may share in common some fundamental features (e.g., the HPA axis is acutely activated by both interoceptive and exteroceptive stressors), interoceptive and exteroceptive models are clearly differential. Each model has its advantages and disadvantages, but it seems that models using exteroceptive stress (Sections 3 and 4) produce more contradictory data than interoceptive stress (Section 5). Though models of exteroceptive stress mimic natural stressogenic factors, including emotional ones, the data show that similar “standard” stress paradigms may be slightly different in different laboratories. In addition, different rodent strains used, different maintaining conditions, and obvious gender dependence of the stress response make a large portion of the published data contradictory and fragmentary. Taking this into account, a future perspective of using stress-related models of depression may be related to investigations of mechanisms contributing to individual differences in vulnerability to stress-induced depression [143, 201, 202] as well as of the impact of parent's exposure to stress on individual's risk for clinical depression in offspring [203]. Models of interoceptive stress (including proinflammatory stressor, particularly systemic LPS administration), though much further from the etiological “human” stressors, seem potentially more “unifiable.” If the goal is to investigate the involvement of NTFs and cytokines in the development of depression-like syndromes, these models deserve more attention and systematic studies.

Discussing therapeutic potential of NTFs in aging and age-associated disorders, Lanni et al. [3] performed classification of various drugs (approved or currently in R and D) having another recognized mechanism of action as NTFs synthesis inducers. The list of NGF, BDNF, and GDNF synthesis inducers includes a number of drugs with antidepressant effects: tricyclic antidepressant Imipramine, selective serotonin reuptake inhibitor Fluoxetine, irreversible monoamine oxidase inhibitor Tranylcypromine, inhibitor of monoamine oxidase Selegiline, antidepressants Biarylpropylsulfonamides (LY392098, LY404187, LY503430), AMPA receptor potentiator Org24448, noradrenaline and serotonin reuptake inhibitors Venlafaxine, Amitriptyline and Clomipramine, adrenergic alpha2-autoreceptors and alpha2-heteroreceptors antagonist Mianserin, and 5-HT2 and 5-HT3 blocker Paroxetine. It is hard to believe that this is just a coincidence. Obviously, effective antidepressants are simultaneously NTFs optimizers in the brain. The NTF systems are closely related to neuroinflammation. NGF regulates a variety of immune functions and BDNF may play a role in the innate and adaptive immune system. The NTFs affect the development and integrity of the noradrenergic, dopaminergic, serotonergic, glutamatergic, and cholinergic neurotransmitter systems. The balance between NTFs support and dysfunction may be at the base of the link between inflammation and neurodegeneration [3]. Changes in NTFs availability inevitably disturb NTFs-mediated signaling and switch on adaptive/compensatory mechanisms, including neuroinflammatory ones. Adaptive during the early phase of stress, the latter may become maladaptive and contribute to the development and progression of depressive state, turning into one of the major mechanisms of its pathogenesis. The abovementioned suggests that the NTFs and cytokine systems in models of depression may be primary targets for both efficient fair experimental studies and development of drugs for treatment of depression states.

## Figures and Tables

**Table 1:** Alterations in the neurotrophic factor system and cytokines in animal models used in translational depression research.

Model	Neurotrophins	References	Cytokines	References
Prenatal stress	BDNF/Bdnf expression ↓ *(amygdala, hippocampus) *	[31–34]	IL-1*β* mRNA ↑* (hippocampus)* IL-10 ↓	[40] [41]
	Methylation of BDNF exon IV ↑	[31–34]		
	m-BDNF/pro-BDNF ↓* (hippocampus) *	[36]		

Early postnatal stress				
(i) Early maternal separation	BDNF ↑, NGF ↑, NT-3 ↑ *(dorsal and ventral hippocampus) *	[55, 58, 60]	IL-1*β* ↑, IL-6 ↑ *(blood)* chemokine ligand 7 ↓, chemokine receptor 4 ↓, IL-10 ↓, IL-1*β* ↓, IL-5 ↓ *(brain) *	[64, 65]
(ii) Maternal deprivation	IGF-1 and IGF-1R mRNAs ↑ *(cerebellum) *	[57]		
	BDNF, TrkB mRNAs and proteins ↑ *(cerebellum) *	[56]		
	NGF, TrkA, p75 NTR mRNAs and protein unchanged *(neocortex) *	[56]		

Social isolation	BDNF mRNA ↓ *(hippocampus) *	[71]	IL-6 ↓, IL-4 ↓, TNF-*α* ↑, IFN*γ* ↑ *(blood) *	[73, 74]

Learned helplessness	BDNF ↓ or no changes *(frontal cortex, hippocampus) *	[95–98, 101]	Data insufficient
	BDNF mRNA ↓ *(hippocampus) *	[97, 98]	
	NGF ↑ *(hippocampus) *	[100]	

Chronic mild stress	BDNF ↓ *(hippocampus, prefrontal cortex)* BDNF mRNA ↓* (hippocampus, hypothalamus)* BDNF mRNA unchanged in hippocampus and amygdala VEGF mRNA ↓ or unchanged hippocampus	[69, 92] [92, 108, 109] [62, 103–105] [114, 115]	IL-1*β* ↑, TNF-*α* ↑, IL-6 ↑ *(blood) *	[79, 81, 82, 86, 87, 89, 90, 93]
			IFN-*γ* ↑, TNF-*α* ↑, IL-6 ↑* (prefrontal cortex) *	[94]
			IL-1*β* ↑, TNF-*α* ↑, IL-10 ↓, IL-4 ↑ mRNAs *(cortex) *	[92]
			IL-1*β* ↑, TNF-*α* ↑, IL-18 ↑, IL-4 ↑ TGF-*β* ↓ mRNAs* (hippocampus) *	[88]
			IL-6 mRNA ↑ *(hypothalamus) *	[93]
			IL-6 unchanged in brain and blood	[86]

Social defeat	BDNF ↓ *(hippocampus) *	[124, 125]	IL-6 ↑ *(hippocampus)* IL-6 mRNA ↑ in brain IL-1*β* unchanged in brain IL-1*β*, TNF-*α* mRNAs unchanged in brain but increased after challenge	[124–126] [127] [125] [126, 127]
	BDNF mRNA ↓* (hippocampus) *	[128, 129]		
	BDNF ↑ *(n. accunbens) *	[131]		
	NGF ↑ *(hippocampus of subordinates)* NGF ↑ *(blood)*	[129]		
	FGF2, FGFR1 mRNAs ↓ *(hippocampus) *	[136]		

Olfactory bulbectomy	BDNF* ↓ (hippocampus) *	[149]	IL-1*β* ↑ *(blood and brain)* TNF-*α* ↑ *(brain) *	[151] [149]
	BDNF mRNA ↑ *(hippocampus) *	[152]		
	NGF mRNA* ↓ (hippocampus) *	[151]		

*Sickness behavior *	Data insufficient		IL-1*β*, IL-6, TNF-*α* mRNAs ↑ *(hypothalamus) *	[172, 173]

*Note.* the data presented in Table 1 were extracted from the papers cited in the review. Most researches in the field are focused on the studies of alterations in the BDNF system and three cytokines, IL-1*β*, IL-6, and TNF-*α*, which are suggested to be principally involved in neuroinflammation associated with depression.

## Abbreviations

ACTH: Adrenocorticotrophic hormone
BrdU: Bromodeoxyuridine
BDNF: Brain-derived neurotrophic factor
CREB: cAMP response element-binding protein
CMS: Chronic mild stress
DCX: Doublecortin
ERK: Extracellular signal-regulated kinase
GDNF: Glial-cell-line-derived neurotrophic factor
GR: Glucocorticoid receptor
FGF: Fibroblast growth factor
FST: Forced swim test
HPA: Hypothalamic-pituitary-adrenal
Iba1: Ionized calcium binding adaptor molecule 1
IGF-I: Insulin-like growth factor-I
IL: Interleukin
IFN: Interferon
LH: Learned helplessness
LPS: Bacterial lipopolysaccharide
LTD: Long-term depression
LTP: Long-term potentiation
NGF: Nerve growth factor
NT-3: Neurotrophin-3
NTFs: Neurotrophic factors
OB: Olfactory bulbectomy
PD: Postnatal day
PNS: Prenatal stress
PTSD: Posttraumatic stress disorder
TGF: Transforming growth factor
TNF-*α*: Tumor necrosis factor-alpha
Trk: Tyrosine receptor kinase
TST: Tail suspension test
VEGF: Vascular endothelial growth factor.
